# Frequency and clinical significance of chromosomal inversions prenatally diagnosed by second trimester amniocentesis

**DOI:** 10.1038/s41598-022-06024-x

**Published:** 2022-02-09

**Authors:** Chih-Wei Chien, An-Shine Chao, Yao-Lung Chang, Kuan-Ju Chen, Hsiu-Huei Peng, Yu-Ting Lin, Angel Chao, Shuenn-Dyh Chang

**Affiliations:** 1 Department of Obstetrics and Gynecology, New Taipei Municipal Tu Cheng Hospital, 6, Sec. 2, Jincheng Road, Tu Cheng, New Taipei City, 236 Taiwan; 2 Department of Obstetrics and Gynecology, Linkou Chang Gung Memorial Hospital and Chang Gung University, 5, Fu-Shin Street, Kwei-Shan, Taoyüan, 333 Taiwan

**Keywords:** Medical research, Molecular medicine

## Abstract

To compare the frequency and clinical significance of familial and de novo chromosomal inversions during prenatal diagnosis. This was a retrospective study of inversions diagnosed prenatally in an Asian population by applying conventional GTG-banding to amniocyte cultures. Data from 2005 to 2019 were extracted from a single-center laboratory database. The types, frequencies, and inheritance patterns of multiple inversions were analyzed. Pericentric variant inversions of chromosome 9 or Y were excluded. In total, 56 (0.27%) fetuses with inversions were identified in the 15-year database of 21,120 confirmative diagnostic procedures. Pericentric and paracentric inversions accounted for 62.5% (35/56) and 37.5% of the inversions, respectively. Familial inversions accounted for nearly 90% of cases, and de novo mutation was identified in two pericentric and two paracentric cases. Inversions were most frequently identified on chromosomes 1 and 2 (16.1% of all inversions), followed by chromosomes 6, 7, and 10 (8.9% of all cases). The indications for invasive testing were as follows: advanced maternal age (67.3%), abnormal ultrasound findings (2.1%), abnormal serum aneuploidy screening (20.4%), and other indications (10.2%). The mode of inheritance was available for 67.9% of cases (38/56), with 89.5% of inversions being inherited (34/38). A slight preponderance of inheritance in female fetuses was observed. Three patients with inherited inversions opted for termination (two had severe central nervous system lesions and one had thalassemia major). Gestation continued for 53 fetuses, who exhibited no structural defects at birth or significant developmental problems a year after birth. Our study indicates that approximately 90% of prenatally diagnosed inversions involve familial inheritance, are spreading, and behave like founder effect mutations in this isolated population on an island. This finding can help to alleviate anxiety during prenatal counseling, which further underscores the importance of parental chromosomal analysis, further genetic studies, and appropriate counseling in cases where a nonfamilial inversion is diagnosed.

## Introduction

Chromosomal inversions constitute a subclass of mutations that involve a change in the orientation of a DNA segment within a chromosome. An inversion is classified as pericentric if the inverted segment includes the centromere (with both arms involved), and it is categorized as paracentric if the two breaks appear on the same side of the centromere (in the same arm). In recent years, many types of chromosomal structural variants have been discovered in the human genome, and their functional effects are gradually being comprehended. However, inversions, especially those mediated by inverted repeats or segmental duplications, are poorly characterized in the literature and difficult to study. Polymorphic inversions constitute structural variants that are difficult to analyze due to their balanced nature and breakpoints being located within complex repeated regions; knowledge of their potential functional effects remains limited^1,2^. Most familial inversions are balanced rearrangements that do not induce abnormal phenotypes or functions in carriers. However, this balanced nature, together with the fact that many of these inversions are mediated by repeats, complicates their analysis. The main inversion-related concern is focused on offspring, where duplications or deletions may arise as a consequence of inversion loops or recombination events^1,3^. Published human genomic information has indicated that structural variation in such inversions is more common than previously estimated, and interest in such variations has been increasing accordingly^1,2,4–6^. Several prenatal cytogenetic analyses have reported an incidence of chromosomal inversion of approximately 1–2/1000^3,7–12^. Until now, the prevalence and nature of chromosomal inversion in the Taiwanese population has not been studied, therefore, our experience offers basic clinical insight on this subject.

## Materials and methods

A retrospective analysis was conducted to examine prenatal inversion diagnoses made from 2005 to 2019 at the cytogenetic laboratory of Chang Gung Memorial Hospital (a tertiary medical center), Linkou, Taiwan. The diagnoses were based on cytogenetic analyses of cultured amniocytes obtained through second-trimester amniocenteses. Conventional karyotyping was conducted using the GTG banding method, in which amniocyte cultures were used to detect chromosomal inversions.

Parental demographic information, antenatal clinical data, and pregnancy outcome data were extracted from medical and birth records. In this study, amniocentesis was primarily indicated in the following situations: advanced maternal age, abnormal maternal serum screening results (risk > 1/270), abnormal fetal ultrasound findings (i.e., presence of fetal anomalies or soft markers), a parental chromosomal anomaly, a family history of chromosomal aberrations, or other nonspecific reasons such as elective performance or anxiety. The data were reviewed to determine the type, frequency, and inheritance pattern of several inversions. Chromosomal variants such as the inversions of chromosomes 9 and Y, double satellites or marked satellites on acrocentric chromosomes, and hyperchromatin on chromosomes 1, 9, and 16 were categorized as normal occurrences and thus excluded from the study.

A prenatal chromosome report, together with a detailed personal and family history; a level II ultrasound examination report; and chromosome studies of both parents were used to provide comprehensive counseling to the participants and their families. Pregnancy outcomes and pediatric follow-up results were obtained from medical records and through telephone interviews. The study followed the tenets of the Declaration of Helsinki and was granted ethical approval by the Chang Gung Memorial Hospital Institutional Review Board (approval number: 202001439B0). Informed consent for chromosome study was obtained from all subjects.

## Results

From 2005 to 2019, 21,120 s-trimester amniocenteses followed by chromosomal aberration analysis were performed at Chang Gung Memorial Hospital. In total, 459 numerical abnormalities and 468 structural aberrations were identified in the 15-year database of confirmatory diagnostic procedures in which amniocentesis was used. As a result, 56 (0.27%) fetuses with inversions were identified. In total, 59 fetuses from 49 mothers were identified, including 5 sets of twin pregnancies and 5 mothers carrying subsequent pregnancies. Common inversions—pericentric inversions on chromosomes 9 and Y—accounted for 84.4% of all inversions (303/[56 + 303]); specifically, 237 chromosome 9 inversions, 65 chromosome Y inversions, and 1 case with both chromosome 9 and Y inversions were identified (Table 1).

**Table 1:** Frequencies and types of chromosomal abnormalities in second trimester amniocentesis, N = 21,120.

Type	Number	Frequency (%)
Normal variant inversions	303	1.43
46,XN,inv(9)(p12q13)	237	1.12
46,X,inv(Y)(p11.2q11.2)	65	0.31
46,X,inv(Y)(p11.2q11.2),inv(9)(p12q13)	1	
Other inversions	56	0.27
**Abnormal karyotype**		
Numerical abnormalities	459	2.17
Structural aberrations	468	2.22
Total	927	4.39

**Figure 1:**
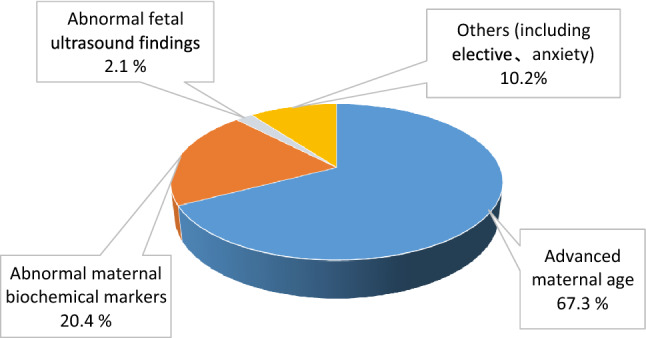
Indications for second trimester amniocentesis in 21,120 patients.

The indications for amniocentesis are presented in Fig. 1. All of the mothers involved in the study agreed to undergo a level II ultrasound examination, and only 32.7% of the mothers declined a parental chromosomal study to determine if their inversions were de novo or hereditary in origin.

Our results revealed that inversions were most frequently identified on chromosomes 1 and 2, with each identified in nine fetuses (16.1%) and accounting for 32.1% of all inversions. The chromosome inv(1)(p13q21) alone was discovered in seven fetuses (12.5%). The distribution and inversion types on all other chromosomes were also analyzed, and no inversions were observed on chromosomes 15, 19, 20, 21, or 22 in our study (Table 2). Pericentric and paracentric inversions accounted for 62.5% and 37.5% of all inversions, respectively (Table 3). The mode of inheritance was available in 67.9% of cases, and 89.5% of inversions were inherited. Although paracentric inversions are considered harmless, de novo mutations warrant special caution. Our study identified four (two pericentric and two paracentric) fetuses who did not have clinical developmental defects, which was determined through close observation over 5 years of postnatal follow-up. The sample had a slight preponderance of maternal inheritance (47.4%) and of female fetuses having inversions (55.4%).

**Table 2:** Distribution of chromosome inversion.

Chromosome	Karyotype	Number	Heredity	% of total
1	46,XN,inv(1)(p22.3p34.1)	1	Paternal	16.1
	46,XN,inv(1)(p36.3q11)	1	De novo	
	46,XN,inv(1)(p13q21)	7	Unknown: 5, Maternal: 2	
2	46,XN,inv(2)(p11.2q13)	5	Unknown: 4, Paternal: 1	16.1
	46,XN,inv(2)(p13q25)	1	Unknown	
	46,XN,inv(2)(p21;q21)	2	Maternal: 1, Paternal: 1	
	46,XN,inv(2)(p25.1q31)	1	Maternal	
3	46,XN,inv(3)(q13.2q27)	1	Paternal	1.8
4	46,XN,inv(4)(q12q21)	1	Paternal	5.4
	46,XN,inv(4)(p14q25)	2	Paternal: 2	
5	46,XN,inv(5)(p12q15.1)	1	Unknown	5.4
	46,XN,inv(5)(q23.2q33.3)	1	Maternal	
	46,XN,inv(5)(p13q22)	1	Maternal	
6	46,XN,inv(6)(p11.2p21.1)	2	Paternal: 2	8.9
	46,XN,inv(6)(q21q25)	1	Paternal	
	46,XN,inv(6)(p12q13)	1	Paternal	
	46,XN,inv(6)(p21.1q15)	1	Maternal	
7	46,XN,inv(7)(q22q34)	3	Unknown: 2, Maternal: 1	8.9
	46,XN,inv(7)(q22q31.3)	2	Maternal: 1 , Paternal: 1	
8	46,XN,inv(8)(q21.2q24.1)	1	Maternal	5.4
	46,XN,inv(8)(q13q21.2)	1	Paternal	
	46,XN,inv(8)(p22q22.3)	1	Paternal	
10	46,XN,inv(10)(p13q11.2)	2	Maternal: 2	8.9
	46,XN,inv(10)(p11.2q22.1)	3	Unknown: 3	
11	46,XN,inv(11)(p11.2q13)	2	Maternal: 2	5.4
	46,XN,inv(11)(p11.2q12)	1	Unknown	
12	46,XN,inv(12)(q21q24.1)	1	De novo	3.6
	46,XN,inv(12)(p13.1q13.1)	1	Unknown	
13	46,XN,inv(13)(q21.2q22)	1	Paternal	3.6
	46,XN,inv(13)(q14.3q21.2)	1	Maternal	
14	46,XN,inv(14)(q22q24.3)	1	De novo	1.8
16	46,XN,inv(16)(p13.1q11.2)	1	Maternal	1.8
17	46,XN,inv(17)(p11.2q21.1)	1	De novo	1.8
18	46,XN,inv(18)(q21.1q23)	3	Maternal: 2, Paternal: 1	5.4
Total		56

**Table 3:** Pregnancy outcomes associated with 56 cases of chromosomal inversion.

Factor	Number (total n = 56)	Percent
**Type**		
Pericentric	35	62.5%
Paracentric	21	37.5%
**Heredity**		
Maternal	18	47.4% (18/38)
Paternal	16	42.1% (16/38)
De novo	4	10.5% (4/38)
Unknown	18	
**Sex**		
Male	25	44.6%
Female	31	55.4%
**Pregnancy outcome**		
Live birth	53	94.6%
Elective termination	3*	5.4%
Fetal death	0	
**Major structural defect/systemic disease**		
Yes*	2	3.6%
No	54	96.4%

*CNS defects in two and one thalassemia major.

The postnatal records revealed that one fetus with anencephaly had a paracentric inversion on chromosome 6, one had beta thalassemia major, and one had a severe spinal anomaly linked to pericentric inversion 10; these pregnancies were all terminated (Table 3). The remaining 53 mothers, who had normal targeted ultrasound examination results, completed their pregnancies successfully and gave birth to newborns with normal development (based on pediatric follow-up conducted for at least 18 months after birth).

## Discussion

Inversions constitute a diverse class of chromosomal mutations. The incidence of prenatally diagnosed inversions in the general population is estimated to be low, affecting only in 1.2-2.5/1000 of the population^3,8–11,13^. In the Taiwanese population, the incidence has been estimated to be 1.92/1000 in the amniocentesis database between 1996 and 2003^14^. The present study revealed a similar incidence of 2.7/1000, with four cases of de novo mutation (Table 2). Similar to other types of mutations, inversions evolve under selection and random drift, particularly those mediated by inverted repeats or segmental duplications, resulting in a spectrum of manifestations ranging from phenotypic silence to neurologic and reproductive consequences. Of the inversions detailed in Table 2, only a small fraction was reported from other ethnic groups or geographic regions. For instance, the frequent inversions were at inv (1)(p13q21), 2(p11q13), inv (2)(p21q21), inv (6)(p12q13), inv (10)(p11.2q22.1) and inv (10)(p13q11.2)^8,9,11,15^. The analysis in the present study may suggest inversions displaying distinct evolution orientation that cause independent gene expressions. In our study group, 95% were Taiwanese, while less than 5 % were aborigines. Because 90% of inversions described in this study were inherited, we presume that the familial inversions are formed in Taiwan, rather than brought by ancestors from other countries. This finding culminates in a conclusion that these inversions spread and that the mutations had a founder effect on this specific island population.

Familial inversions may remain undetected unless major shifts in landmark bands are observed. Fluorescence in situ hybridization (FISH) and array-CGH techniques are useful for detecting and characterizing chromosomal rearrangements by revealing cryptic microdeletions or microduplications at or near the breakpoints^15^. Phenotypic alterations and mental retardation likely result from the dysfunction of specific genes located at one or both breakpoints, from a positional effect of adjacent chromatin, or from complex trans effects.

Most pericentric inversions affect the pericentric region of chromosomes 1, 2, 3, 5, 9, 10, 16, and Y; and are considered nonpathological polymorphisms. The relatively common inv(2)(p11.2q13) has exhibited several reported exceptions, but none were observed in the present study^3,25–27^. Pericentric inversions can produce recombinant gametes; however, few meiotic segregation studies have explored the relationship between the frequency of recombinants and inverted segment size^1,3^. Specific pericentric inversions, such as the pericentric inversion of chromosome 9 and various polymorphisms of the Y chromosome, are considered common pericentric variants, with varying incidences among distinct population groups. The incidence of inv (9) is highest and lowest among those of African (3.57%) and Asian ethnicities (0.26%), respectively. The highest incidence of the Y chromosome is noted in the Asian population (3.37%)^15^. Pericentric inversions of chromosomes also frequently involve autosomal chromosomes, such as chromosomes 1, 5, 8, 11, and 12^16,17^. In the present study, the prenatal detection rates of chromosome 9 and Y inversion were 1.12% and 0.31%, respectively; these results represent lower incidences than those reported in the literature^16–19^. A key reason for these differences was the population base. Most studies have suggested possible associations between chromosomal inversions and particular pathologies (e.g., infertility, habitual abortion, autism or schizophrenia, developmental delay, and speech disorders)^3,16,20^ that appear in adults or specific patient groups. No notable family history of neuropsychological disorder was reported by any of the parents examined in the present study.

Paracentric inversions reportedly occur in all human chromosomes, but they are most common on chromosomes 1, 3, 5, 6, 7, 11, and 14 and less common on chromosomes 4, 16, 17, 18, 19, 20, 21, 22, and Y^12,21^. Paracentric inversions of 1q are also less common than those of 1p. However, patterns of inversions vary with ethnicity^1,3,22–24^. The noteworthy findings of the present study were that paracentric inversions occurred most frequently on chromosomes 7 and 18 and that pericentric inversions occurred most frequently on chromosomes 1 and 2 (Table 2). Nearly two-thirds of the inversions were pericentric, which corresponds to findings reported in the literature^1^. The inversions diagnosed in our cases did not appear to affect all chromosomes equally. Inversions on chromosomes 1, 2, 6, 7, and 10 were noted in 58.9% of the fetuses, and no inversions were identified on chromosomes 9, 15, 19, 20, 21, or 22. Although no paracentric inversions with recombinants were identified in this study, counseling should be offered if and when they occur, such as in cases afflicted by inv(7)(q13.31q31.33), inv(9)(p13p24), inv(9)(q22.1q34.3), inv(14)(q24.2q32.3), inv(17)(p11.2p13), inv(18)(q12.1q23), inv(18)(q21.1q22.3), and inv(18)(q21.32q23), because multiple unpredictable and unbalanced chromosome products have been reported and may be involved ^1,3,12,21^.

Studies have indicated that although most inversions are harmless, mental retardation, infertility, and miscarriage occur in some cases. The site and size of the inverted segment are reportedly related to the risks of partial trisomy and monosomy (due to meiosis recombination and the likelihood of early spontaneous loss). Thus, larger pericentric inversions are more likely to result in the birth of a child with aneuploidy, whereas smaller pericentric inversions are more likely to be associated with recurrent pregnancy loss due to the duplication or deletion of large chromosome segments^1,3^. In the present study, two (out of 49) sets of parents had a history of recurrent miscarriage and subfertility and had been seeking assistance from fertility clinics.

Among the four de novo inversions observed, two were paracentric, namely inv(12)(q21q24.1) and inv(14)(q22q24.3), and two were pericentric, namely inv(1)(p36.3q11) and inv(17)(p11.2q21.1). Genetic counseling was conducted with the support of several online databases (i.e., http://www.ncbi.nlm.nih.gov, Google Scholar, Scopus, PubMed, and InvFEST)^25^ and English-language articles published up to December 2019. The aforementioned four inversions were evaluated but were without correlated phenotypes or neurological deficits. In these cases, the parents all underwent a chromosomal analysis and a family history review, and all decided to continue with their pregnancy after receiving normal detailed ultrasound examination results. None of the de novo inversions had yielded clinically significant adverse outcomes at a mean follow-up of 5 years after childbirth. Nevertheless, nonfamilial inversions may carry a risk of phenotype anomalies due to nonpaternity, gonadal mosaicism, or a de novo nature. In the present analysis, all of the involved parents had clinically healthy infants and were without a family history of chromosomal aberrations. They declined our requests to participate in molecular studies.

Ultrasound is a vital aspect of antenatal genetic counseling. A risk of an abnormal phenotype arising from the disruption of dosage-sensitive or regulatory genes at specific inversion breakpoints was reported^25^. Two CNS defects with 46,XX,inv(6)(p11.2p21.1) and 46,XX,inv(10)(p11.2q22.1) and one case of thalassemia with 46,XY,inv(12)(p13.1q13.1) were observed in the present study. No previous study has established any connection between CNS defects and chromosomal inversions. Hence, the occurrence of these two cases was likely coincidental. The single case of thalassemia major was also not related to the pericentric inversion.

Notably, we discovered that inversions had a slight female hereditary predominance (55.4%, Table 3). However, the distribution of maternal and paternal inheritance was almost equal. Although sex ratio shifts should be a random phenomenon, a recent study hypothesized that they are an aspect of evolution resulting from the genetic recombination that occurs during meiosis I^3^. A multitude of potential reasons might explain why most inversions are more prevalent in women than in men. Future studies of X chromosomes may identify new genes and provide further insight into the complex mechanisms underlying mutations.

A limitation of this study was that no cases were subjected to molecular analyses, which limited possible findings such as cryptic microdeletions or microduplications that specifically require this technology. In recent years, molecular techniques have become useful for detecting and characterizing chromosomal rearrangements by revealing cryptic microdeletions or microduplications at or near the breakpoints in particular cases^3,26–28^. Three-color FISH techniques should be included in comprehensive parental studies with offspring having de novo insertions or a family history of suspected submicroscopic inversion, especially for microdeletion or microduplication syndromes. Array-based CGH can be conducted to explore the loss or gain of chromosomal material more precisely than by using conventional chromosome analyses. However, the parents in 30% of our cases declined to undergo chromosomal analysis because they did not have a specific family history. Proper counseling and financial support through governmental aid may help affected parents.

## Conclusion

Prenatally diagnosed chromosomal anomalies present challenges in genetic counseling. Our study indicates that nearly 90% of prenatally diagnosed inversions involve familial inheritance for both common and uncommon inversions, are formed in Taiwan, rather than brought by ancestors from other countries. Moreover, parental chromosomal analysis is essential in cases where an uncommon inversion is diagnosed. The inversions examined in this study did not present clinical phenotypic significance in the pediatric period. Overall, the information from this study can help alleviate anxiety in parents receiving prenatal counseling.
